# Predictive Modeling of Cycloplegic Refraction Using Non-Cycloplegia Ocular Parameters With Emphasis on Lens-Related Features

**DOI:** 10.1167/tvst.14.10.3

**Published:** 2025-10-01

**Authors:** Qiang Su, Bei Du, Bingqin Li, Chen Yang, Yicheng Ge, Haochen Han, Chea-Su Kee, Wenxue Li, Ruihua Wei

**Affiliations:** 1 Tianjin Key Laboratory of Retinal Functions and Diseases, Tianjin Branch of National Clinical Research Center for Ocular Disease, Eye Institute and School of Optometry, Tianjin Medical University Eye Hospital, Tianjin, China; 2 School of Optometry, The Hong Kong Polytechnic University, Hong Kong SAR, China; 3 North Sichuan Medical College, Nanchong, China

**Keywords:** optometry, refractive error, machine learning, cycloplegia

## Abstract

**Purpose:**

The study aimed to develop a predictive model for refraction after cycloplegia by leveraging non-cycloplegia ocular parameters and focusing on lens-related features.

**Methods:**

A total of 153 children 4 to 15 years old were enrolled in this study. This study randomized gender distribution. Sex, age, intraocular pressure (IOP), refraction before and after cycloplegia, and optical biometry (OB) parameters were collected. Four prediction models for spherical refraction were developed: a control group without lens-related features and three experimental groups incorporating lens-related features. Features such as lens diopter, anterior surface curvature radius, and lens thickness played significant roles. The models were evaluated using statistical measures: mean square error (MSE), Root mean square error (RSME), Mean absolute error (MAE) and r-square (*r*^2^). Least absolute shrinkage and selection operator (LASSO) regression and the L1 regularization term were used for feature screening and machine learning for extreme gradient enhancement. The extreme gradient boosting (XGBoost) method was used to develop the model.

**Results:**

The predictive model incorporating lens-related features demonstrated superior performance in estimating refraction after cycloplegia compared to the model without such features. Among the models with lens-related features, the IOL of contact lens algorithm (IOL_cl_) group exhibited the highest efficacy, boasting an *r*^2^ of 0.964, MSE of 0.241, RMSE of 0.472, and MAE of 0.307.

**Conclusions:**

The study provided valuable insights into developing a robust predictive model for refraction after cycloplegia, emphasizing the importance of lens-related features and the morphological changes in the crystalline lens during accommodation.

**Translational Relevance:**

This predictive model has potential advantages in avoiding complications associated with cycloplegia and can be widely applied for clinic vision screening in optometry.

## Introduction

Myopia stands as one of the prevailing refractive errors, constituting an irreversible refractive ocular ailment that is characterized by a substantial incidence and swift progression, particularly among children around the world. Projections indicate an anticipated escalation by the year 2050, reaching 49.8% for myopia and 9.8% for high myopia.^1^ Consequently, the expeditious identification, timely diagnosis, and prompt intervention in cases of myopia are of paramount significance. Research has demonstrated that the change of diopter before and after cycloplegia is negatively correlated with age.^2^ Cycloplegia refers to the paralysis of the ciliary muscle of the eye, resulting in a loss of accommodation. It is often induced to accurately measure refractive errors without the eye's accommodation. This correlation—particularly pronounced in younger age groups—results in overestimation of myopia or underestimation of hyperopia with non-cycloplegia refraction as a benchmark.^3^^–^^5^ Hence, it is imperative to advocate for the utilization of cycloplegia refraction, acknowledged as the gold standard in clinical practice.^6^^–^^8^ However, negative effects of cycloplegia medications can last for several hours (tropicamide) or as long as several weeks (atropine), making the process time consuming to perform.^9^ Other challenges, such as clinic time, render widespread application in school-based vision screening impractical. For example, teenage subjects can complete a regular examination without cycloplegia in under 30 minutes; however, if cycloplegia is required, they will spend at least 1 hour at the optometry center, and the negative effects can last several hours at home. Therefore, this predictive model offers the advantage of reducing both clinic time and complications associated with cycloplegia, such as photophobia due to pupil dilation, blurred vision from accommodation paralysis, potential allergies, and headaches that may occur during the cycloplegia procedure.^10^ Consequently, some parents express concern and may reject the cycloplegia process.

Numerous prior studies have explored factors such as sex, age, visual acuity, refractive error, axial length, axial length/radius of corneal curvature, accommodation lag, and other optical biometric parameters to formulate predictive models for estimating refractive error with cycloplegia. Leveraging spherical equivalent, age, and uncorrected visual acuity, Sankaridurg et al.^2^ initially constructed a linear model linking the spherical equivalent before and after cycloplegia, achieving an *r*^2^ of 0.91. Independently, teams led by Magome et al.^11^ and Wang et al.^12^ developed cycloplegia spherical (S) refraction and spherical equivalent prediction models using multivariable regression models with root mean square error (RMSE) values of 0.87 and 0.64, respectively. Additionally, incorporating visual acuity, intraocular pressure (IOP), lag of accommodation, gaze deviation, spherical and cylindrical refraction, and optical biometric parameters, Du et al.^13^ formulated a cycloplegia spherical equivalent prediction model using a machine learning approach with a RMSE of 0.50 and an *r*^2^ of 0.93. A comparison of the specifications between this study and other references and a comprehensive assessment of the characteristics and performance metrics are presented in Table 1. Although these studies have yielded commendable outcomes, it is crucial to highlight that a comprehensive understanding of the predicted physical models has not been fully attained.

**Table 1.** Comparison of the Specifications Between This Study and Other References

Ref.	Mean ± SD	Ages (Y)	Features	Performance
Sankaridurg et al.^2^	−0.74 ± 1.79 D^a^	4–15	SE, age, UCVA	*r* ^2^ = 0.91
	(−11.00, +8.00)			
Magome et al.^11^	0.25 ± 2.32 D^b^ (−6.00, +8.75)	2–9	Gender, age, AL, ACD, LT, Km, AST	*r* ^2^ = 0.92, RMSE = 0.87
Wang et al.^12^	−1.12 ± 1.98 D^a^ (—)	5–18	Gender, age, GW, SE, AL/CR, UCVA, IOP	*r* ^2^ = 0.92, RMSE = 0.64
Du et al.^13^	−1.13 ± 1.58 D^a^	6–18	VA, IOP, LOA, GD, S, C, OB	*r* ^2^ = 0.93, RMSE = 0.50
	(—)			
This work	−1.54 ± 2.17 D^b^ (−7.50, +10.50)	4–15	Gender, age, S, IOL, VCD, IOP, OB, lens-related features	*r* ^2^ = 0.96, RMSE = 0.47

ACD, anterior chamber depth; AL, axial length; AL/CR, axial length/radius of corneal curvature; AST, astigmatism; C, cylindrical refraction; GD, gaze deviation; GW, glasses-wearing status; IOP, intraocular pressure; Km, mean keratometry; LOAcc, lag of accommodation; LT, lens thickness; OB, optical biometry parameters; RMSE, root mean square error; S, spherical refraction; SE, spherical equivalent; UCVA, uncorrected visual acuity (min, max), the interval range between minimum and maximum values; VA, visual acuity; VCD, vitreous chamber depth.

a Spherical equivalent.

b Spherical refraction.

In response to this challenge, the purpose of this study was to develop a prediction model focusing on lens-related features to predict refraction of cycloplegia more accurately and to use clinical experiments to verify the physical model used in this study. Prior to performing cycloplegia, clinicians can use our predictive model to estimate the cycloplegic refraction value. If the difference between the predicted value and the non-cycloplegic value is minimal, cycloplegia may not be necessary, thereby reducing clinic time and alleviating cycloplegia complications.

## Methods

The study encompassed the collection of data pertaining to sex, age, IOP, non-cycloplegia refraction, optical biometry (OB) parameters, and cycloplegia refraction from all subjects. These examinations are part of routine assessments conducted in optometry clinics for school-aged children, and the data were directly accessible. Cycloplegia was induced using 1% cyclopentolate, with each eye receiving 1 drop at 5-minute intervals for a total of three drops. Subsequently, participants rested for 30 minutes with pressure on the inner canthus of both closed eyes. Following the cycloplegia, refraction measurements were taken using the same autorefraction instrument.

**Figure 1.**
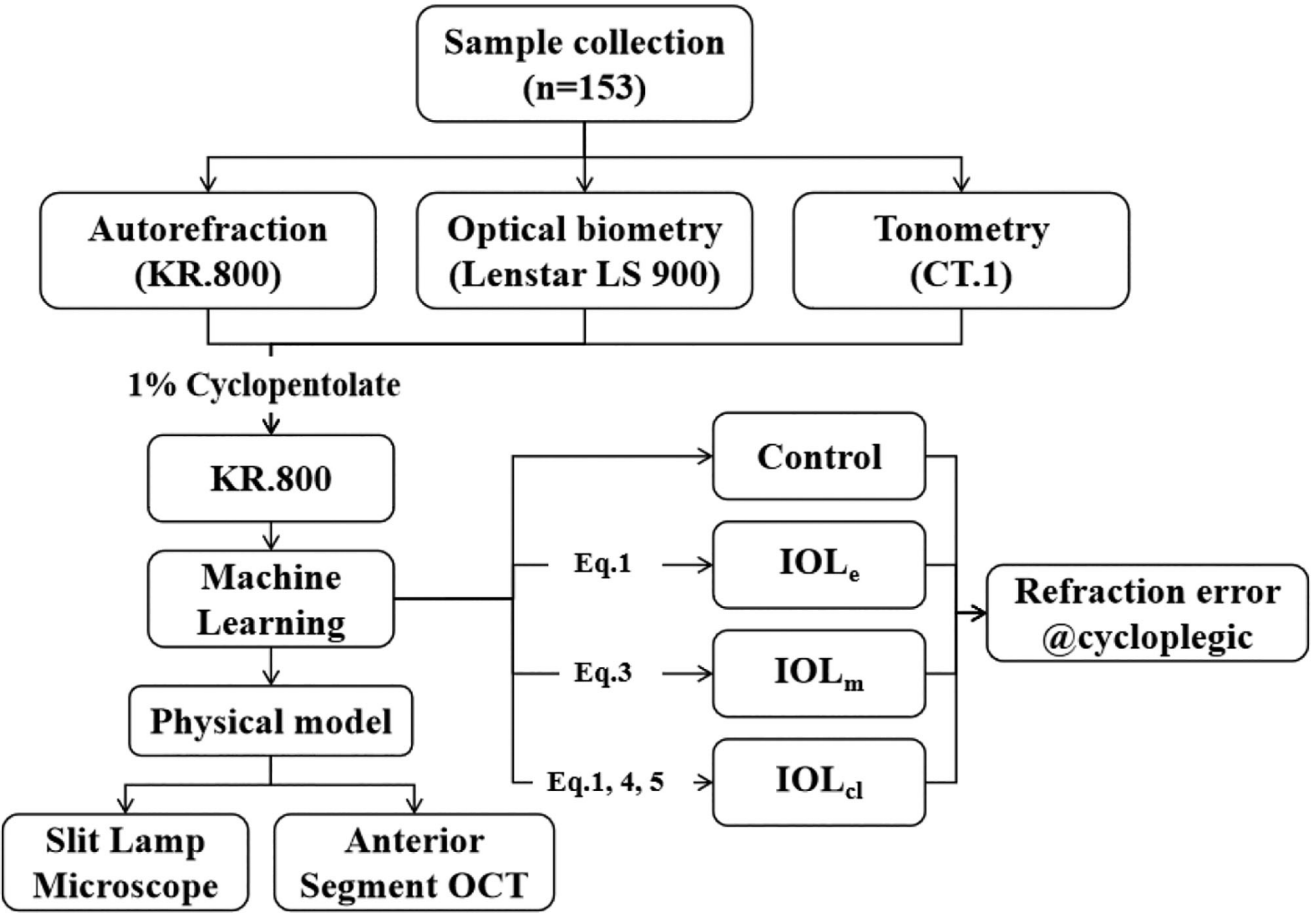
Flowchart of the predictive model based on the lens features.

Refraction measurements before and after cycloplegia were conducted by using autorefraction (KR-800; Topcon Healthcare, Tokyo, Japan). During the measurement, the subjects were instructed to relax and focus on the distant target to facilitate accommodation relaxation. At least three measurements were taken for each eye to make sure that the difference among each measurement was less than 0.25 diopters (D). The instrument average value was recorded as the objective refraction. Optical biometry parameters, including axial length (AL ), central cornea thickness (CCT), anterior chamber depth (ACD), lens thickness (LT), and keratometry readings (K1, K2, and Km), were measured by using a Lenstar 900 (Haag-Streit, Köniz, Switzerland). At least three measurements were taken for each eye, and the average value was obtained with a change of AL and Km less than 0.02 mm and 0.50 D, respectively. IOP was measured by a non-contact CT-1/CT-1P computerized tonometer (Topcon Healthcare), with three measurements taken for each eye and the average results recorded. The research flowchart is depicted in Figure 1.

The model utilizes routinely measured non-cycloplegic parameters: demographic data (age, gender) from clinical records, spherical and cylindrical refraction via autorefraction (Topcon KR-800), and optical biometric parameters (e.g., AL, ACD, K) obtained from devices such as the Lenstar 900. Direct lens parameters (e.g., anterior/posterior lens curvature radius) are not required for clinical application. Instead, lens-related features are derived algorithmically from the routine inputs using the SRK/T formula (for workflow, see Fig. 1). In this article, these error metrics, such as mean absolute error (MAE), RMSE, and mean square error (MSE), are crucial for evaluating the accuracy and reliability of the predictive model. The clear presentation of these metrics allows for a transparent evaluation of the findings of this study and makes comparison with other studies^11^^–^^13^ possible.

The implementation of this study followed the tenets of the Declaration of Helsinki. This study was approved by the ethics committee of Tianjin Medical University Eye Hospital (approval ID: 2020KY-39). Only children whose legal guardians provided written informed consent were enrolled.

Due to the unavailability of lens parameters in routine optometry examinations, this study employed the SRK/T formula IOL model as a substitute for crystalline lens diopter in the analysis. The SRK/T formula, a third-generation IOL calculation method, is known for its high precision in predicting refractive outcomes in clinical practice.^14^ Studies have demonstrated that it provides satisfactory accuracy in calculating refractive error in eyes.^15^^,^^16^ Furthermore, research has shown that there is no statistically significant difference in mean absolute prediction error among the OKULIX, SRK/T, and Hoffer Q formulas, indicating that all three perform with comparable accuracy.^17^ The SRK/T formula calculates IOL diopter based on commonly measured parameters such as keratometry (Km), AL, and ACD, all of which can be easily obtained through routine clinical examinations. The IOL of emmetropia eye algorithm (IOL_e_) diopter could be calculated by the SRK/T formula, as depicted in Equations 1 and 2:

(1) $$\begin{array}{c} IOL=\frac{1000n_{a}\left(n_{a}\cdot r-\Delta n\cdot LOPT\right)}{\left(LOPT-ACD\right)\left(n_{a}\cdot r-\Delta n\cdot ACD\right)} \end{array}$$

(2) $$\begin{array}{c} LOPT=AL+0.65696-0.02029AL \end{array}$$

where *n*_a_ = 1.336 is the refractive index of the aqueous and vitreous, Δ*n* = 0.333 is the refractive index difference, *r* is the mean corneal radius of curvature (mm), and *LOPT* is the corrected axial length (mm). As per Equation 1, it is important to note that the IOL_e_ diopter represents the value corresponding to an emmetropic eye, which did not accurately reflect the actual refractive error of participants. Therefore, when updating the calculated method of the IOL_e_ group to the IOL of myopia eye algorithm (IOL_m_) group and IOL of contact lens algorithm (IOL_cl_) group, it was imperative to incorporate the refractive error of the participants into the calculation.

In incorporating the actual IOL diopter of subjects with refractive error, the IOL_m_ was computed using the near-use formula for the retained refraction, as given by the SRK/T formula:

(3) $$\begin{array}{ccc} & & \;IOL_{m}\\ & & =\frac{\begin{array}{c} 1000n_{a}\{n_{a}\cdot r-\Delta n\cdot LOPT-0.001SE\\ \;[12(n_{a}\cdot r-\Delta n\cdot LOPT)+LOPT\cdot r]\} \end{array}}{\begin{array}{c} \left(LOPT-ACD\right)\{n_{a}\cdot r-\Delta n\cdot ACD-0.001SE\\ \;[12(n_{a}\cdot r-\Delta n\cdot LOPT)+ACD\cdot r]\} \end{array}} \end{array}$$

For the IOL_cl_ group, the contact lens diopter (*F*_c_) could be calculated using the conversion formula between the contact lens and the objective refraction by the KR-800 lens in Equation 4. The *F*_c_ diopter was then superimposed on the cornea, resulting in the corrected corneal curvature radius (*r*_cl_) as Equation 5. Subsequently, the actual IOL_cl_ diopter, considering refractive error, was calculated by Equation 1.

(4) $$\begin{array}{c} F_{c}=\frac{SE}{1-0.012SE} \end{array}$$

(5) $$\begin{array}{c} r_{cl}=\frac{337.5}{K_{m}+F_{c}} \end{array}$$

In the IOL_e_, IOL_m_, and IOL_cl_ groups, the refractive parameters of the lens anterior surface, encompassing lens anterior surface refraction (*F_a_*) and lens anterior surface radius of curvature (*r*_a_), were computed using the thick lens formula $F_{a}=\frac{F_{p}-IOL}{(LT/n)-1}$ and the refraction formula $r_{a}=\frac{1000(n-n_{a})}{F_{a}}$, where *F*_p_ = 9.26 D represents the refraction of the lens posterior surface from the model eye of Bennett and Rabbetts,^18^ and *n_a_* = 1.40 is the refractive index of lens.

The control group model incorporated traditional eye features, but the experimental group models added lens-related features to the control group. The experimental group models three distinct IOL-derived parameters (IOL_e_, IOL_m_, IOL_cl_), each calculated via incremental refinements of the SRK/T formula (Equations 1–5), to quantify lens contributions and predict cycloplegic refraction. The features included in each model are specified in Table 2.

**Table 2.** Features Included in Each Prediction Model

Control	IOL_e_	IOL_m_	IOL_cl_
Gender			
Age	Control	Control	Control
S	+	+	+
IOP	ACD	ACD	ACD
AL	LT	LT	LT
CCT	IOL_e_	IOL_m_	IOL_cl_
IOP/CCT	*F_a_e_*	*F_a_m_*	F*_a_cl_*
AL/CCT	AL/ACD	AL/ACD	AL/ACD
K1	AL/LT	AL/LT	AL/LT
K2	AL/VCD	AL/VCD	AL/VCD
Km	*r_a_e_*	*r_a_m_*	*r_a_cl_*
*r*	LT/*r_a_e_*	LT*/r_a_m_*	LT/*r_a_cl_*
AL/CR	ACD/*r_a_e_*	ACD*/r_a_m_*	ACD/*r_a_cl_*
VCD			

These IOL variants, alongside lens anterior curvature (*r_a_*) and interaction terms (such as LT/*r_a_*), were input into the XGBoost algorithm as lens-related features. LASSO regression first screened features in Table 2 and modeled nonlinear relationships between these parameters (e.g., IOL_cl_ + AL/ACD + age) to predict cycloplegic spherical refraction*.*

For further analysis purposes, the subjective refraction of cylinder (C) diopter was converted into J0 and J45 by the power-vector notation:^6^^,^^19^^,^^20^$J0=-\frac{C}{2}\cos2\alpha ,J45=-\frac{C}{2}\sin2\alpha$, where the J0 and the J45 represent the two cylindrical vectorial components with C ≤ 0.00 D in the calculation.

### Statistical Analysis

Across the entire cohort of 306 eyes, the diopters of cycloplegia compared with the non-cycloplegia refraction were compared and respectively categorized into ΔS and ΔC. For ΔS, the 95% limits of agreement (LOAs) ranged from 0.28 to 0.40 D (*r*² > 0.97); the diopter change within the ± 0.25-D range only accounted for 67% of the total, and maximal ΔS was more than +2.00 D. In comparison, the ΔC group exhibited LOAs from −0.07 to −0.002 D (*r*² > 0.99). Notably, the ΔC groups were predominantly concentrated within the ± 0.25-D range which is consistent with the typical clinical measurement error of 0.25 D, as indicated in Table 3. The C magnitude was likely so small that it would not result in any noticeable or meaningful difference in vision correction or treatment, which is similar to the conclusions of other studies.^6^^,^^11^^,^^21^^,^^22^ For the predictive model, the prediction model developed in this study focuses solely on predicting S after cycloplegia, as it accurately predicts meaningful outcomes even when some statistically significant findings, such as C differences, are clinically negligible.

**Table 3.** Statistical Analysis of S and C Errors Before and After Cycloplegia

	Non-Cycloplegia		Cycloplegia		Paired *t*-Test		
Parameter	Mean	SD	Mean	SD	MD ± SD	*t*	*p*
S (D)	−1.53	2.17	−1.19	2.41	0.34 ± 0.53	11.19	<0.001
C (D)	−0.87	0.75	−0.91	0.77	−0.03 ± 0.30	−2.13	0.044

MD, mean difference.

**Figure 2.**
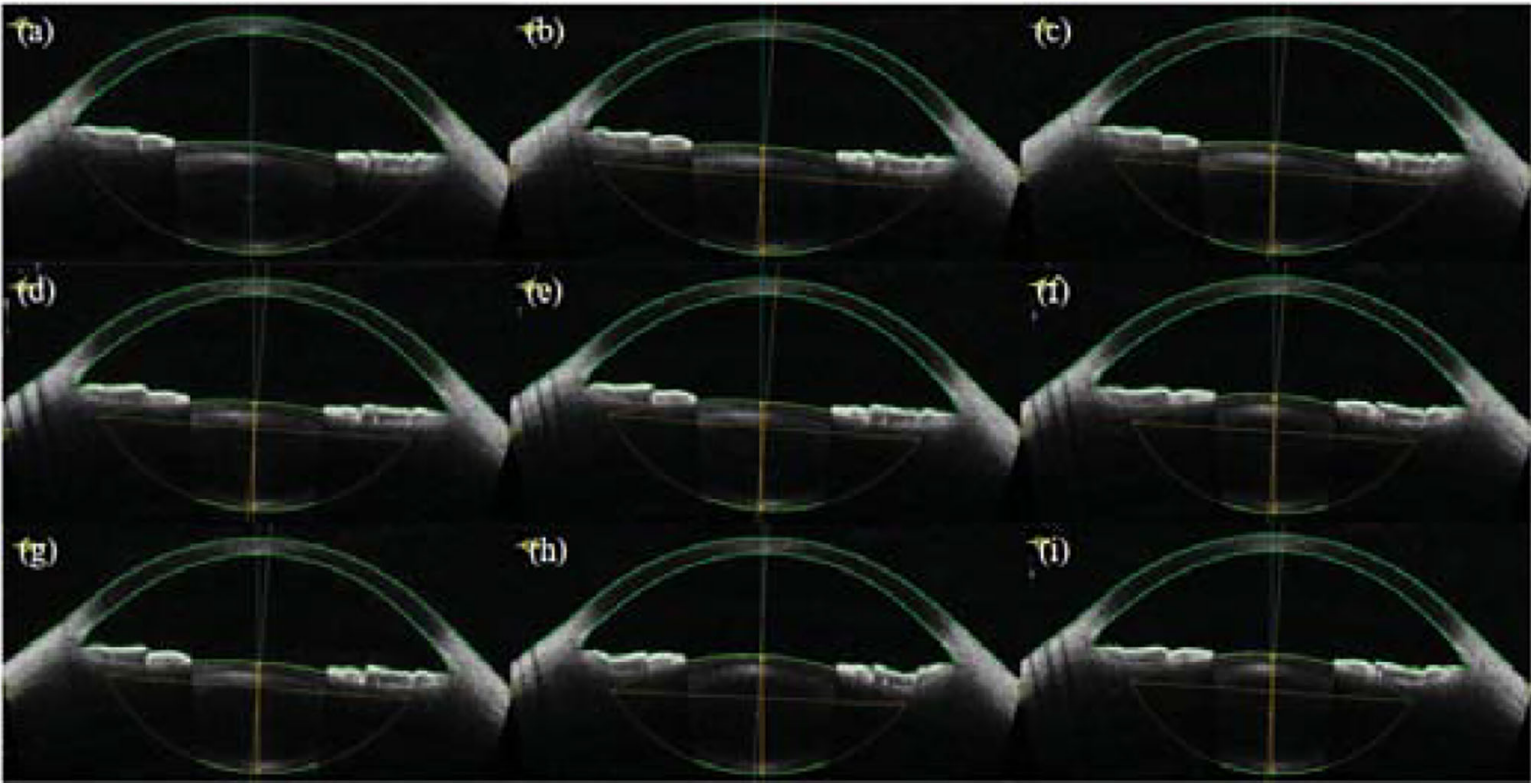
(**a**–**i**) Typical anterior segment images captured by AS-OCT across a range of accommodations, from minimal (**a**) to maximal (**i**).

The lens has become a prominent feature in ocular predictive modeling, which contains a wealth of information.^23^ It is noteworthy that the changes in refraction induced by cycloplegia can be attributed to the lens. Lens parameters were acquired using anterior segment optical coherence tomography (AS-OCT, CASIA2; Tomey Corporation, Aichi, Japan) under varying accommodation diopters, as depicted in Figure 2. The AS-OCT measurements provided detailed parameters of the lens along the horizontal direction (180°), including the anterior surface curvature radius (ACR) of the lens, the posterior surface curvature radius (PCR) of the lens, and LT, precisely obtained under different accommodations. The lens diopter was calculated using the refraction formula and the thick lens formula.^24^^,^^25^ As accommodation increased, it is noteworthy that the changes in ACR were significantly larger than those for PCR, especially in low accommodation diopters. The refractive change after cycloplegia typically remains below 1.50 D in most subjects.^4^^,^^5^^,^^26^ Compared to non-cycloplegia, lens parameters under cycloplegia primarily changed in ACR, ACD, and LT, whereas the PCR remained constant, as supported by several previous studies.^24^^,^^27^^−^^31^

**Figure 3.**
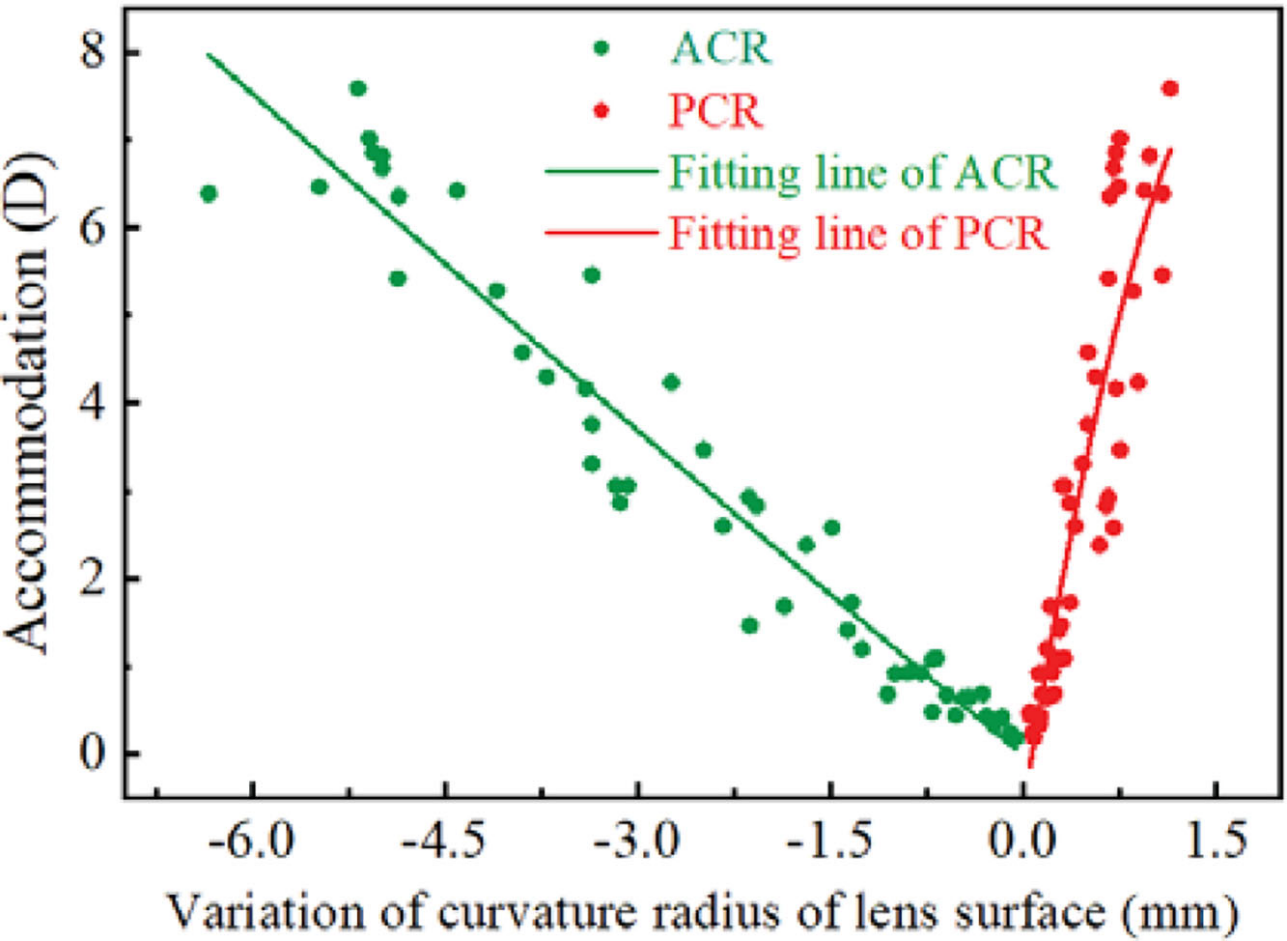
Scatterplots show the changes in anterior (ACR, *green*) and posterior (PCR, *red*) lens curvature radii (mm) during accommodation, as observed across eight subjects by AS-OCT. Quadratic function fitting produces solid curves that closely align with these data points (Equation 6 for ACR, Equation 7 for PCR). Notably, at low accommodation diopters (<1.50 D), the change in PCR remained nearly negligible.

Figure 3 illustrates the relationship between the changes in ACR and PCR with stepwise accommodation stimulation across eight subjects (16 eyes). The ACR and PCR values were referenced without accommodation (ACC) as the origin point of coordinates. The mean ± SD changes in radius per diopter were 0.83 ± 0.25 mm/D for ACR and 0.19 ± 0.08 mm/D for PCR. Quadratic function fitting produced solid curves for these data points, showing a highly significant relationship (*r*^2^ = 0.94 for ACR, *r*^2^ = 0.82 for PCR), expressed in Equations 6 and 7 as follows:

(6) $$\begin{array}{ccc} Acc\left(D\right) & = & 0.01\times \Delta ACR^{2}-1.21\\ & & \times \Delta ACR-0.02 \end{array}$$

(7) $$\begin{array}{ccc} Acc\left(D\right) & = & -2.40\times \Delta PCR^{2}+9.30\\ & & \times \Delta PCR-0.60 \end{array}$$

Considering the small change in diopter after cycloplegia (typically less than 1.50 D), Equations 6 and 7 were inverted to obtain the slopes of ACR (|*k*_ACR_| ≈ 1.21) and PCR (|*k*_PCR_| ≈ 9.30). It was observed that under the condition of cycloplegia, the change in ACR surpassed that of PCR significantly. This further supported the validity of the physical model, suggesting that the lens diopter after cycloplegia primarily originates from ACR.

## Results

In this investigation, four prediction models for S were established, comprised of one control group and three experimental groups. To assess the impact of lens-related features on predicting S in cycloplegia, the performance of the control and experimental models was compared. Subsequently, feature screening was conducted utilizing least absolute shrinkage and selection operator (LASSO) regression, a technique that eliminates unimportant features by applying L1 regularization, resulting in the reduction of coefficients for irrelevant or redundant features to zero. The extreme gradient boosting (XGBoost) algorithm, which offers higher predictive accuracy and more reliable feature importance assessment compared to traditional machine learning models such as logistic regression and support vector machines, was selected to model the screened features in this study. A grid search was combined with a fivefold cross-validation method to determine the optimal hyperparameter configuration. The key parameters of learning rate, maximum depth of the tree, subsample ratio, and regularization parameters were carefully adjusted to ensure that the performance of the model on the training set could be well generalized to the test set. In this study, the contribution of lens-related features to predicting S in cycloplegia was validated by comparing the MSE, RMSE, MAE, and *r*^2^ values of the four models.

Eighty percent of the dataset, after missing value interpolation was completed, was randomly allocated as the training set for model training, and the remaining 20% was designated as an independent test set to assess the predictive efficiency of the model. The performance of the control model and the experimental models to predict S diopter after cycloplegia are presented in Table 4. The percentage of samples with a difference between the predicted value and the true value within 0.50 D can then be determined. The control group reached 87.1% accuracy, and the IOL_e_ and IOL_m_ groups achieved 88.71%. IOL_cl_ demonstrated the highest accuracy at 90%.

**Table 4.** Performance of Four Prediction Models

Group	MAE	MSE	RMSE	*r* ^2^
Control	0.350	0.301	0.531	0.933
IOL_e_	0.316	0.252	0.482	0.964
IOL_m_	0.312	0.242	0.473	0.963
IOL_cl_	0.307	0.241	0.472	0.964

In the control group, the performance without the inclusion of lens-related features aligned with the findings of previous studies, particularly the work by Du et al.^13^ (MSE = 0.226, RMSE = 0.50, MAE = 0.351, and *r*^2^ = 0.93), compared with MSE = 0.301, RMSE = 0.531, MAE = 0.350, and *r*^2^ = 0.933 in this work. In comparison with the control group, the performance of the experimental groups was significantly enhanced, signifying the pivotal role of lens parameters in predicting S after cycloplegia. Particularly, the IOL_cl_ group exhibited the best performance in predicting S, with MSE = 0.241, RMSE = 0.472, MAE = 0.307, and *r*^2^ = 0.964.

Paired *t*-tests were employed to assess the significance of differences between the predicted and true values in each group and are detailed in Table 5. The results indicated that the disparities between predicted and true values were statistically significant in the control group (*P* = 0.04), with mean 95% LOAs of 0.13 ± 0.98. However, in the three IOL groups, paired *t*-test results demonstrated no statistically significant differences (*P* > 0.05), suggesting a closer alignment between predicted and true values. Notably, the IOL_cl_ group exhibited the most accurate predictions, with mean LOAs of 0.07 ± 0.84. Hence, it is evident that the predictive effect of the IOL_cl_ group model is the closest to the true values.

**Table 5.** Statistical Analysis of Predictive and True Values for Four Groups

	Predictive Value		True Value		Paired *t*-Test			
Groups	Mean	SD	Mean	SD	MD ± SD	*t*	*p*	LOAs
Control	−1.46	1.84			0.13 ± 0.50	2.06	0.04^*^	0.13 ± 0.98
IOL_e_	−1.41	1.94	−1.33	1.99	0.08 ± 0.43	1.54	0.13	0.08 ± 0.84
IOL_m_	−1.41	1.90			0.08 ± 0.44	1.40	0.17	0.08 ± 0.86
IOL_cl_	−1.40	1.90			0.07 ± 0.43	1.33	0.19	0.07 ± 0.84

* *P* < 0.05.

## Discussion 

To evaluate the consistency of the model's performance across different age groups, study subjects were divided into two subgroups based on age: 4 to 8 years old (*n* = 110) and the 9 to 15 years (*n* = 196). We evaluated the performance of the model separately within each subgroup, including MAE, MSE, RMSE, and *r*^2^, as shown in Table 6. The analysis results show that the performance of the model in predicting refractive status is relatively stable in both subgroups. The proposed method has good applicability in a wide age range. This finding enhances the generalization of predictive model in practical clinical applications.

**Table 6.** Age Subgroup Analysis of XGBoost Model for IOL_cl_ Group

	Mean ± SD			
Age	MAE	MSE	RMSE	*r* ^2^
4–8 y	0.406 ± 0.036	0.353 ± 0.159	0.582 ± 0.121	0.941 ± 0.021
9–15 y	0.334 ± 0.063	0.331 ± 0.246	0.523 ± 0.190	0.939 ± 0.029

In this study, because of its strong performance and interpretability, XGBoost was used for small and medium sample sizes. To further illustrate results, the performance of a predictive model was compared with other machine learning algorithms, including linear regression and support vector machines, as shown in Table 7.

**Table 7.** Results of Other Modeling Methods for IOL_cl_ Group

	Mean ± SD			
Model	MAE	MSE	RMSE	*r* ^2^
LR	0.322 ± 0.021	0.519 ± 0.129	0.334 ± 0.157	0.953 ± 0.034
SVM	0.252 ± 0.039	0.494 ± 0.195	0.316 ± 0.182	0.938 ± 0.041

LR, logistic regression; SVM, support vector machine.

Although neural networks provide high modeling flexibility and the potential to capture complex nonlinear relationships, they usually require larger datasets and are easier to overfit in smaller samples. In preliminary comparison, the neural network model failed to fit. Compared with these alternatives, the model selected exhibited more consistent performance, lower computational complexity, and greater transparency, attributes that are particularly valuable in clinical settings where model interpretability is critical.

The reported MAE of 0.307 D demonstrates high clinical utility, as prediction errors within ±0.50 D align with accepted thresholds for refractive prescriptions and myopia progression monitoring. Clinically, predictions within this range may allow clinicians to bypass cycloplegia for stable cases, applying cycloplegic approaches for discrepancies > 0.50 D. Current school-based myopia screening predominantly utilizes non-cycloplegia methods, which can lead to inaccuracies in detecting pseudo-myopia. This prediction model enables a more accurate representation of population-level true myopia rates, reducing diagnostic biases inherent to non-cycloplegic approaches. This predictive model demonstrated excellent performance and can be used to monitor epidemiological trends with great reliability by balancing efficiency and safety.

By reducing reliance on cycloplegia, this model addresses two critical challenges: (1) minimizing complications such as photophobia and prolonged blurred vision, and (2) enabling efficient, non-invasive screenings in school or resource-limited settings. For example, clinicians could use routine autorefraction and biometry data to estimate cycloplegic refraction, applying cycloplegia for subjects with significant discrepancies (>0.50 D).

This section discusses in detail why the anterior surface of the lens mainly changes during accommodation. The crystalline lens should be treated as a thick lens, and the position of the image principal plane is determined by factors such as the refractive power of the anterior and posterior lens surfaces (*F_a_* and *F_p_*, respectively), lens thickness (*LT*), and the refractive index of the lens (*n_L_*), as expressed in $p^{'}=-n^{'}\frac{F_{a}}{F}\frac{LT}{n_{L}}$, where the value of *p*′ is the distance between the rear vertex of the lens to the image principal point in Descartes’ rule of signs.

To intuitively illustrate the position change of the image principal plane during the accommodation, the reference point is shifted from the rear vertex to the center point of lens. In the Descartes coordinate system, with no accommodation assumed, the coordinate origin *O* is positioned at the center point of the lens, and the distance from *O* to the image principal plane *P*′ is denoted as *OP*′. The *OP*′ value equals the sagittal height value of the posterior surface of the lens plus the *p*′ value, as represented in $OP^{'}=r_{p}-\sqrt{{r_{p}}^{2}-{\left(\frac{d_{L}}{2}\right)}^{2}}+p^{'}$, where *r_p_* and *d_L_* are the PCR and the thickness of lens, respectively.

**Figure 4.**
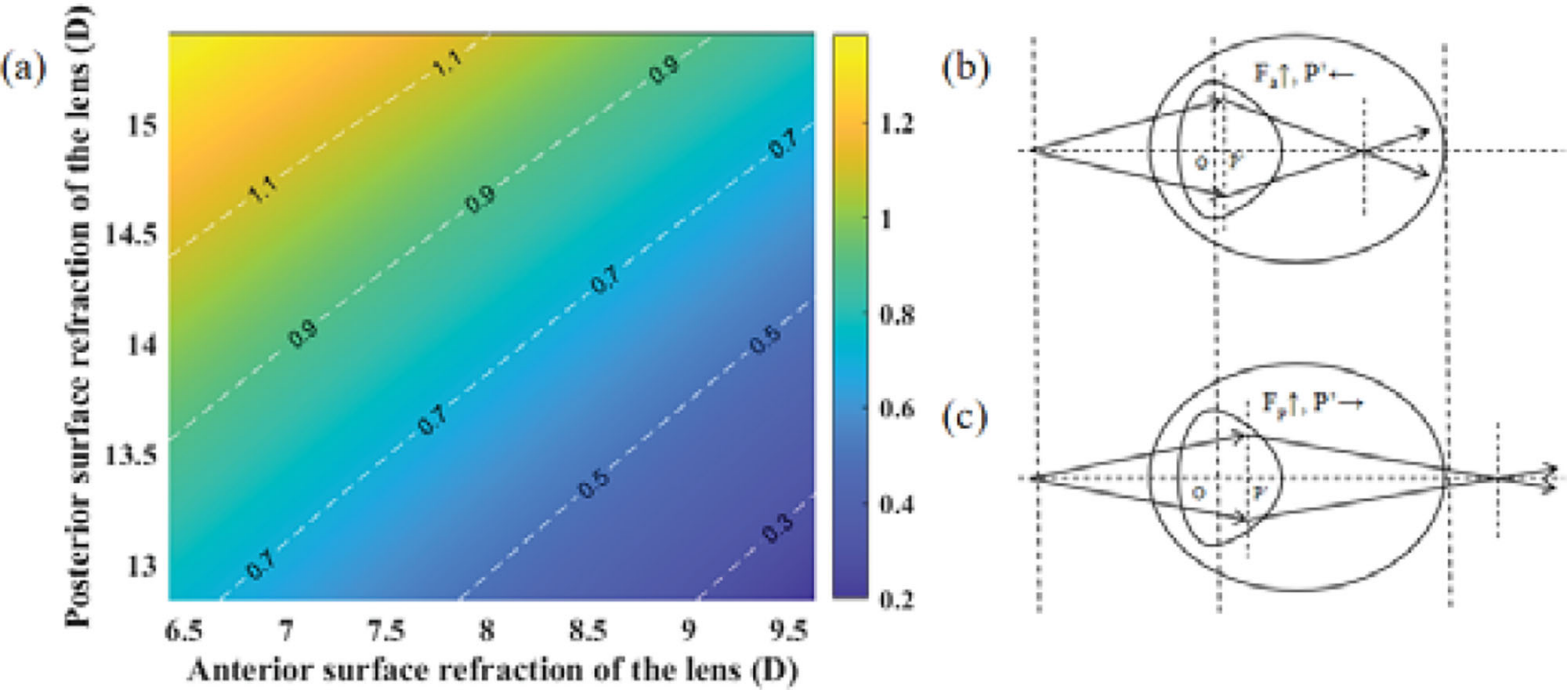
(**a**) The relationship among the *OP*′ (color bar value) and the diopter of the anterior (*F_a_*) and posterior (*F_p_*) surfaces of the lens. (**b**, **c**) Movement direction of the image principal plane with the changes in refraction of *F_a_* and *F_p_*.

Based on data sourced from Dubbelman et al.,^29^ Figure 4a illustrates the correlation between the *OP*′ value and the refraction of *F_a_* and *F_p_*. It was observed that, as the *F_a_* refraction increased, the *OP*′ value decreased, leading to a leftward displacement of the image principal plane, as depicted in Figure 4b. This shift toward the cornea resulted in a decrease in overall eye diopter, necessitating less accommodation for focusing onto the retina. Conversely, as shown in Figure 4c, as the refraction of *F_p_* increased, the *OP*′ value increased, causing a rightward shift of the image principal plane to the retina. This displacement led to an increase in overall eye diopter. Consequently, the ciliary muscle must exert greater force to realign focus onto the retina during accommodation.

The human eye tends to favor the path of least resistance or effort. The ciliary muscles will use relatively minimal force to change ACR and focus onto retina, aligning with the principle of optimization within the visual system. Consequently, during the cycloplegia process, there may be a preference for changes in the ACR due to the lower force requirements. This analysis offers insights into the physical mechanisms involved in changes in the anterior surface of the lens and their influence on the overall diopter of the eye during accommodation.

The study has limitations, notably the relatively small sample size compared to other studies, which may result in more predictive error for cases with larger or smaller refractive errors, reduced generalizability of the findings, or potential biases in the machine learning methodology. Regarding the lack of data, we will continue to conduct a multicenter study in Chongqing, and Hong Kong and other regions are being considered. We hope to obtain broader and more comprehensive data and improve the external validity and universality of the study.

This study focused on developing a refraction prediction model for cycloplegia, utilizing non-cycloplegia ocular parameters. The inclusion of lens-related features significantly improved the performance of predictive models for cycloplegic refraction, indicating the importance of lens parameters in predictive models. Analysis of lens parameters revealed that changes in the ACR are notably greater than those in the PCR during cycloplegia. This reaffirms the validity of the physical model, suggesting that cycloplegic refraction primarily originates from alterations in the ACR. Overall, the findings underscore the importance of considering lens-related features in predictive modeling and provide valuable insights into the optical processes underlying accommodation.
